# Supplementary material for: Rapid turnover of life-cycle-related genes in the brown algae
Source: Genome Biol. 2019 Feb 14;20:35. doi: 10.1186/s13059-019-1630-6 (PMC6374913; doi:10.1186/s13059-019-1630-6)
Supplement: Supplementary file 3 — Protein alignments, corresponding Gblocks html file and CDS sequences for the lineage conserved gametophyte-biased genes. (ZIP 2109 kb) [file 13059_2019_1630_MOESM3_ESM.zip › Con_GA_alignments/Ec-12_003960.aa_ali.fasta-gb.htm]

Ec-12\_003960.aa.fasta.trans.aa\_ali.fasta


## Gblocks 0.91b Results

Processed file: **Ec-12\_003960.aa.fasta.trans.aa\_ali.fasta**  
Number of sequences: **2**  
Alignment assumed to be: **Protein**  
New number of positions: **283** (selected positions are underlined in blue)

```
                         10        20        30        40        50        60
                 =========+=========+=========+=========+=========+=========+
Ec-12_003960     MEGLPQNYRDNITIDNGVEIVWVNQKGQVIPKDKAGAASSAVEDDADVVVGMPRVASIRK
MSTRG.11389.1    ------------------------------------------------------------
                                                                             


                         70        80        90       100       110       120
                 =========+=========+=========+=========+=========+=========+
Ec-12_003960     SAVETELNNSSLPASPGGTRRTSVAGTVSPSPQLIGAWFTNGTDTGGEGAPTSPAGSTGP
MSTRG.11389.1    ------------------XRRISVAGTVSPSPQLIGAWFTNGPDGGGGGAPGSPAGSTGP
                                    #########################################


                        130       140       150       160       170       180
                 =========+=========+=========+=========+=========+=========+
Ec-12_003960     PDTEDDDADPEGHPCMALEPYAREMYNTVRKGLLGKKRTPMETLLSWKGKQITVPLTIAC
MSTRG.11389.1    PETEDDDADPDGHPCMALEPYARDMYNTVRKGLLGKKRTPMETLLSWKGKQITAPLTIAC
                 ############################################################


                        190       200       210       220       230       240
                 =========+=========+=========+=========+=========+=========+
Ec-12_003960     NQAKAVSSECVACFGDIMVYMGDKKAKESGMQLIKSLHTRLLASTQEFRDEIICQVCKQT
MSTRG.11389.1    SQNRSMSSECVSWFGDIMVYMGDKKGKESGMQLTKSLQTRLLASDQDFRDEIFCQVCKQT
                 ############################################################


                        250       260       270       280       290       300
                 =========+=========+=========+=========+=========+=========+
Ec-12_003960     TSNPDANSDLRGWQLILSILAVATPSKGLIPCFRKHCELGPVGSIPQTYAQYALSALHKE
MSTRG.11389.1    TSNPDANSDLRGWQLLLSILAVATPSTALVPFFRKHCEFSPVGSIPQTYAQYALSALNKE
                 ############################################################


                        310       320       330       340       350       360
                 =========+=========+=========+=========+=========+=========+
Ec-12_003960     SSLAPRKAAPCDVEIMALAQLSAVPVLVRLQDGNKVEVMADSWTSVTDLEKQIASKIGLK
MSTRG.11389.1    SCLAPRKATPCDVEIMALVQLSAVPVLVRLQDGNKVEVMADSWTSVTDLEKQVCTR----
                 ####################################################        


                        370       380       390       400       410       420
                 =========+=========+=========+=========+=========+=========+
Ec-12_003960     CCTAFTVFEATNTDGQRALRAKERILDTVAQWRTQAAATSGVKKFQLLFKVRLHLRPDDD
MSTRG.11389.1    ---------------------------TVIQTSVAAA-----------------------
                                            ##########                       


                        430       440       450       460       470       480
                 =========+=========+=========+=========+=========+=========+
Ec-12_003960     DLAGIKLTYLQAVQDLCDGVYMVSTEDSVQLAAFQLQASRGDYDKKNCTAGSLEKELTTL
MSTRG.11389.1    ------------------------------------------------------------
                                                                             


                        490       500       510       520       530       540
                 =========+=========+=========+=========+=========+=========+
Ec-12_003960     VPPQILAQPEGDEFRGRKDLANKILAYYMMSLKGISKKEARALFMGLMRENPSFGRAYFD
MSTRG.11389.1    ------------------------------------------------------------
                                                                             


                        550       560       570       580       590       600
                 =========+=========+=========+=========+=========+=========+
Ec-12_003960     GLSQEKPVVVGIGADGVQFLDATDGDRPVLAEYGFDSIGSFVCSDEETAVMTLAAREGEQ
MSTRG.11389.1    ------------------------------------------------------------
                                                                             


                        610       620       630       640       650       660
                 =========+=========+=========+=========+=========+=========+
Ec-12_003960     TSFVKPTAILIQSAQASQMKSLVHAYMVRYILPRRKKKEEGEKAKTVNKETDVVPFATDG
MSTRG.11389.1    ------------------------------------------------------------
                                                                             


                 
                 =
Ec-12_003960     A
MSTRG.11389.1    G
```

```
Parameters used
Minimum Number Of Sequences For A Conserved Position: 2
Minimum Number Of Sequences For A Flanking Position: 2
Maximum Number Of Contiguous Nonconserved Positions: 8
Minimum Length Of A Block: 10
Allowed Gap Positions: None
Use Similarity Matrices: Yes
```

```
Flank positions of the 2 selected block(s)
Flanks: [80  352]  [388  397]  

New number of positions in Ec-12_003960.aa.fasta.trans.aa_ali.fasta-gb:  283  (42% of the original 661 positions)
```
